# Whole brain radiation therapy for patients with brain metastases: survival outcomes and prognostic factors in a contemporary institutional series

**DOI:** 10.1007/s00066-024-02275-x

**Published:** 2024-08-12

**Authors:** Anna Estermann, Chiara Schneider, Frank Zimmermann, Alexandros Papachristofilou, Tobias Finazzi

**Affiliations:** 1 Clinic of Radiotherapy and Radiation Oncology, University Hospital Basel, Petersgraben 4, 4031 Basel, Switzerland; 2 Department of Radiation Oncology, Kantonsspital Baden, Baden, Switzerland

**Keywords:** Brain metastases, Whole brain radiotherapy, Whole brain radiation therapy, WBRT

## Abstract

**Purpose:**

To study survival outcomes and prognostic factors in patients undergoing whole brain radiation therapy (WBRT) for brain metastases in the contemporary setting.

**Methods:**

Patients undergoing WBRT from 2013–2021 were retrospectively included in an ethics-approved institutional database. Patient and treatment characteristics were assessed, including patient age, primary tumor histology, Karnofsky Performance Status (KPS), extracranial disease, as well as WBRT dose. Overall survival (OS) was calculated from onset of WBRT using the Kaplan-Meier method.

**Results:**

A total of 328 patients (median age 63 years) were included. Most patients (52%) had ≥ 10 brain metastases, and 17% had leptomeningeal disease. WBRT was delivered with 10 × 3 Gy (64%), 5 × 4 Gy (25%), or other regimens (11%).

Median follow-up was 4.4 months (range, 0.1–154.3), and median OS was 4.7 months (95%CI, 3.8–6.0). OS differed between histologies (*p* = 0.01), with the longest survival seen in breast cancer (median 7.7 months). Patients with KPS of 90–100 survived for a median of 8.3 months, compared to 4.1 months with KPS 70–80, and 1.7 months with KPS < 70 (*p* < 0.01).

Multivariate analyses revealed that KPS had the largest impact on survival. Patients who received a WBRT dose of ≥ 30 Gy also had a reduced risk of death (HR 0.45; *p* < 0.001). Survival differed between subgroups reclassified according to the Rades scoring system (*p* < 0.01).

**Conclusion:**

Survival outcomes of patients undergoing WBRT in the contemporary era appear comparable to historical cohorts, although individual patient factors need to be considered. Patients with otherwise favorable prognostic factors may benefit from longer-course WBRT.

## Introduction

Brain metastases (BM) are a common occurrence in patients with cancer, and a significant cause of morbidity and mortality. The relevance of BM is increasing due to advances in cancer detection and treatment, which have increased the life expectancy of patients, who may be more likely to develop BM over the course of their disease [1]. Out of all cancer patients, 20–40% will develop BM, which most commonly occur in patients with lung cancer, followed by breast cancer [2, 3]. Most patients with BM have a poor prognosis, with early studies having reported a median survival of approximately 1 month in patients who were left untreated [4, 5]. Current treatment options include whole brain radiation therapy (WBRT), stereotactic radiotherapy including stereotactic radiosurgery (SRS), neurosurgical resection, systemic anti-cancer therapies, dexamethasone, best supportive care (BSC) or a combination of the above [6, 7]. Both initial treatment methods and overall survival (OS) of patients with BM have changed over the last decades. An analysis of patients treated between 1986–2020 revealed that the use of SRS as well as systemic treatment had increased, whereas a decline in neurosurgical resections was observed. Median OS improved from 5 to 7 months over this time period [8].

As a standard treatment for patients with multiple BM, WBRT aims to improve neurological symptoms and quality of life (QoL), prevent additional symptoms and prolong survival [9]. In the past, the toxicity of WBRT appeared less relevant due to the poor life expectancy of patients with BM, as well as the lack of effective treatment alternatives. This has changed due to improvements in OS for many patients with BM, for whom the negative effects of WBRT have gained significance [10, 11]. Acute side effects, which are mostly temporary, may include headaches, fatigue, nausea, erythema, and loss of hair. Late side effects may persist, and this includes neurocognitive decline as the most feared complication after WBRT. Potential side effects must be weighed against the benefits of WBRT in the palliative setting. A randomized study in non-small cell lung cancer (NSCLC) patients with BM who were unsuitable for surgery or SRS showed no difference in OS when WBRT was added to BSC (including dexamethasone) [7]. Despite known limitations of the study [12], this questions the clinical benefit of WBRT especially in patients with a very poor prognosis, and emphasizes the need for individualized assessment in this setting.

A variety of scoring systems have been developed to predict survival outcomes in patients with BM. Recursive partitioning analysis (RPA) was the first scoring system to be widely used in clinical practice. Published in 1997, the system differentiated three classes, with RPA class 1 patients having the best and RPA 3 the poorest prognosis. The classes were primarily based on the Karnofsky Performance Status (KPS) of the patients [13]. Graded Prognostic Assessment (GPA) is another index that has been used to predict the survival of patients with BM. First published in 2008, the score has since been revised to generate diagnosis-specific (DS-)GPA indices, and to incorporate molecular markers [6, 14–16]. Other scoring systems include Score Index for Radiosurgery (SIR), Basic Score for Brain Metastases (BSBM) and the Golden Grading System (GGS), which estimates survival of patients treated with SRS [17–19]. In 2008, Rades et al. published a different scoring system that specifically estimated survival of patients with BM who were treated with WBRT. Their analysis identified four prognostic factors, which were used to calculate four prognostic groups (A-D): age, KPS, the presence of extracranial metastases, and interval between tumor diagnosis and WBRT [20, 21]. The Rades score can be used to estimate survival after WBRT, and identify patients who may be better treated with short-course (e.g. 5 × 4 Gy) rather than longer course WBRT. However, the Rades score was based on a retrospective analysis of patients undergoing WBRT between 1992 and 2005. Since then, stereotactic radiotherapy techniques have been widely adopted, including SRS as a now common approach for patients with multiple BM. Furthermore, the landscape of systemic treatment options has evolved significantly. Both the characteristics and treatment outcomes of patients undergoing WBRT are therefore likely different in current practice, which questions the validity of prognostic scores. This study aims to investigate survival outcomes of patients undergoing WBRT in the contemporary era, which may also support clinical decision making in this palliative setting.

## Methods

This retrospective study included patients with brain metastases undergoing WBRT from 2013–2021 at the University Hospital of Basel in Basel, Switzerland. The project was approved by the Ethics Committee of Northwestern and Central Switzerland. No funding was received for the planning or conduct of this study.

Patients with brain metastases were identified by scanning the department’s radiation oncology information system MOSAIQ (Elekta, Sweden) for diagnoses with an ICD-10 code of C79.3 (secondary malignant neoplasm of brain and cerebral meninges). Patient demographics and clinical parameters were collected from electronic medical records. This data was anonymised using an external data catalogue, and stored on a study-specific Castor EDC platform (Castor, USA). All cases were manually reviewed, with exclusion of patients who did not receive WBRT (e.g. patients who underwent SRS only, or those who received prophylactic rather than therapeutic WBRT).

All patients had undergone WBRT on an Elekta Synergy (Elekta, Sweden) linear accelerator using either 3D-conformal radiotherapy (3DCRT) or volumetric modulated arc therapy (VMAT) with 6 MV photons. The recorded patient and treatment characteristics included factors such as patient age, KPS, primary tumor histology, number of brain metastases, extracranial tumor burden, as well as WBRT dose and fractionation. A focal boost to one or multiple metastases was delivered on an individual basis (e.g. in fitter patients, or when lesions were considered to be symptomatic). Magnetic resonance (MR) and/or computed tomography (CT) imaging of the brain was available in all cases. Survival outcomes were obtained from hospital records, including correspondence with external institutions, and directly from family physicians in selected cases.

OS was calculated from onset of WBRT using the Kaplan Meier (KM) method. A multivariate Cox regression analysis was performed to calculate the hazard ratio (HR) of death after WBRT, using the following variables: sex, age group (≤ 50, 51–70, > 70 years), KPS (< 70, 70–80, 90–100), primary tumor type, controlled primary (e.g. after surgical resection), extracranial metastases, leptomeningeal disease, and WBRT dose applied (< 30 Gy or ≥ 30 Gy; excluding patients who stopped treatment early due to clinical deterioration). In addition, patients were classified according to the Rades Scoring System based on age, KPS, extracranial metastases at the time of WBRT, and interval from tumor diagnosis to WBRT [20]. Statistical analyses were performed using RStudio Version 2023.03.0 + 386 (RStudio, USA).

## Results

A total of 328 patients were identified and included in the analysis. The characteristics of these patients are summarized in Table 1*. *Patients were a median of 63 years (range, 29–85) old at time of WBRT. The proportion of male and female patients was 44% (*n* = 145) and 56% (*n* = 183), respectively. The most common primary tumor histology was NSCLC (41%), followed by breast cancer (22%), small cell lung cancer (SCLC; 13%) and melanoma (9%). Less common primary tumors (all with ≤ 10 cases included) were prostate (3%), esophageal and gastric (2%), gynecological (2%), colorectal (1%), head and neck (1%), and other including undifferentiated histologies (5%). In patients with NSCLC, targetable mutations in the epidermal growth factor receptor (EGFR) and anaplastic lymphoma kinase (ALK) genes were known to be present in 8% and 4% of cases, respectively. In patients with breast cancer, human epidermal growth factor receptor 2 (HER2) mutations were present in 27% of cases, whereas 22% had triple-negative disease.

**Table 1:** Characteristics of patients who underwent WBRT for brain metastases

		Patients (n = 328)
*Sex*	Male	145 (44%)
	Female	183 (56%)
*Age, median (range), y*		63 (29–85)
*Karnofsky Performance Status*	< 70	58 (18%)
	70–80	170 (52%)
	90–100	100 (30%)
*Primary tumor histology*	Non-small cell lung cancer	135 (41%)
	Breast	73 (22%)
	Small cell lung cancer	42 (13%)
	Melanoma	30 (9%)
	Other	48 (15%)
*Number of brain metastases*	≤ 3	79 (24%)
	4–9	77 (23%)
	≥ 10	172 (52%)
*Leptomeningeal disease*	Yes	56 (17%)
	No	272 (83%)
*Extracranial disease*	Yes	262 (80%)
	No	66 (20%)
*Primary tumor controlled*	Yes	159 (48%)
	No	169 (52%)
*WBRT regimen*	10 × 3 Gy	210 (64%)
	5 × 4 Gy	82 (25%)
	6 × 4 Gy	12 (4%)
	Other	24 (7%)
*Boost to macroscopic disease*	Yes	37 (11%)
	No	291 (89%)

*Abbreviations: WBRT* whole brain radiation therapy

The initial treatment intent, at time of first cancer diagnosis, was curative in 145 patients (44%), and palliative in 183 patients (56%). The median KPS at time of WBRT was 80 (range, 30–100), and extracranial disease was present in 80% of patients. Most patients had ≥ 10 brain metastases (*n* = 172; 52%), with the remaining patients having either 4–9 (*n* = 77; 23%) or ≤ 3 metastases (*n* = 79; 24%). Evidence of leptomeningeal disease (with or without parenchymal metastases) was present in 56 patients (17%).

The most common WBRT dose-fractionation scheme was 30 Gy in 10 fractions (*n* = 210; 64%), followed by 20 Gy in 5 fractions (*n* = 82; 25%), and 24 Gy in 6 fractions (*n* = 12; 4%). The remaining 24 patients (7%) were treated with individual dose-fractionation schemes (total dose 18–46 Gy in 4–23 fractions). A focal boost to macroscopic disease was delivered in 37 patients (11%). Patients treated in ≥ 10 fractions had a better KPS than those treated in < 10 fractions (*p* < 0.01), although age was not different between these groups (*p* = 0.21). WBRT was completed as scheduled by 302 (92%) patients. In the remaining 26 patients (8%), WBRT was stopped early due to clinical deterioration or patient choice.

**Fig. 1:**
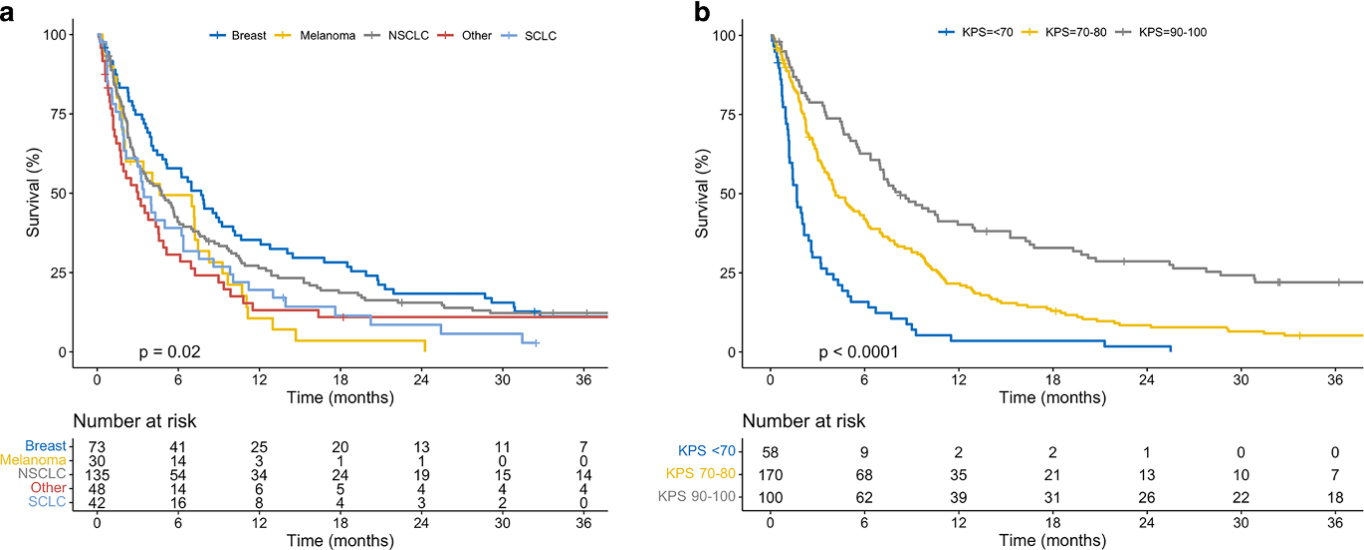
Kaplan-Meier estimates of overall survival following WBRT for brain metastases, stratified by primary tumor type (left panel) and KPS (right panel). More favorable survival outcomes were observed in patients with breast cancer, as well as in those with a good performance status. *Abbreviations: KPS* Karnofsky Performance Status,* NSCLC* non-small cell lung cancer, SCLC small cell lung cancer,* WBRT* whole brain radiation therapy

Median follow-up was 4.4 months (range, 0.1–154.3 months). The KM estimates of survival are shown in Fig. 1. Median OS for the entire cohort was 4.7 months (95%CI, 3.8–6.0) after WBRT. OS differed between primary tumor type (*p* < 0.01; Fig. 1a), with the longest survival seen in patients with breast cancer (median 7.7 months; 95%CI, 5.1–10.2). In comparison, patients undergoing WBRT for metastases of NSCLC (the largest subgroup) had a median OS of 4.8 months (95%CI, 3.2–6.1). Patients with SCLC and melanoma had a median OS of 3.5 months (95%CI, 2.1–6.4) and 4.6 months (95%CI, 2.0–9.2), respectively, whereas patients with other histologies had a median OS of 3.0 months (95%CI, 1.7–4.9). OS also differed between patients stratified by KPS (*p* < 0.01; Fig. 1b). Patients with a KPS of 90–100 survived for a median of 8.3 months (95%CI, 7.0–13.0), compared to 4.1 months (95%CI, 3.5–6.1) in patients with KPS 70–80, and 1.7 months (95%CI, 1.2–2.6) in patients with KPS < 70 (*p* < 0.01).

**Fig. 2:**
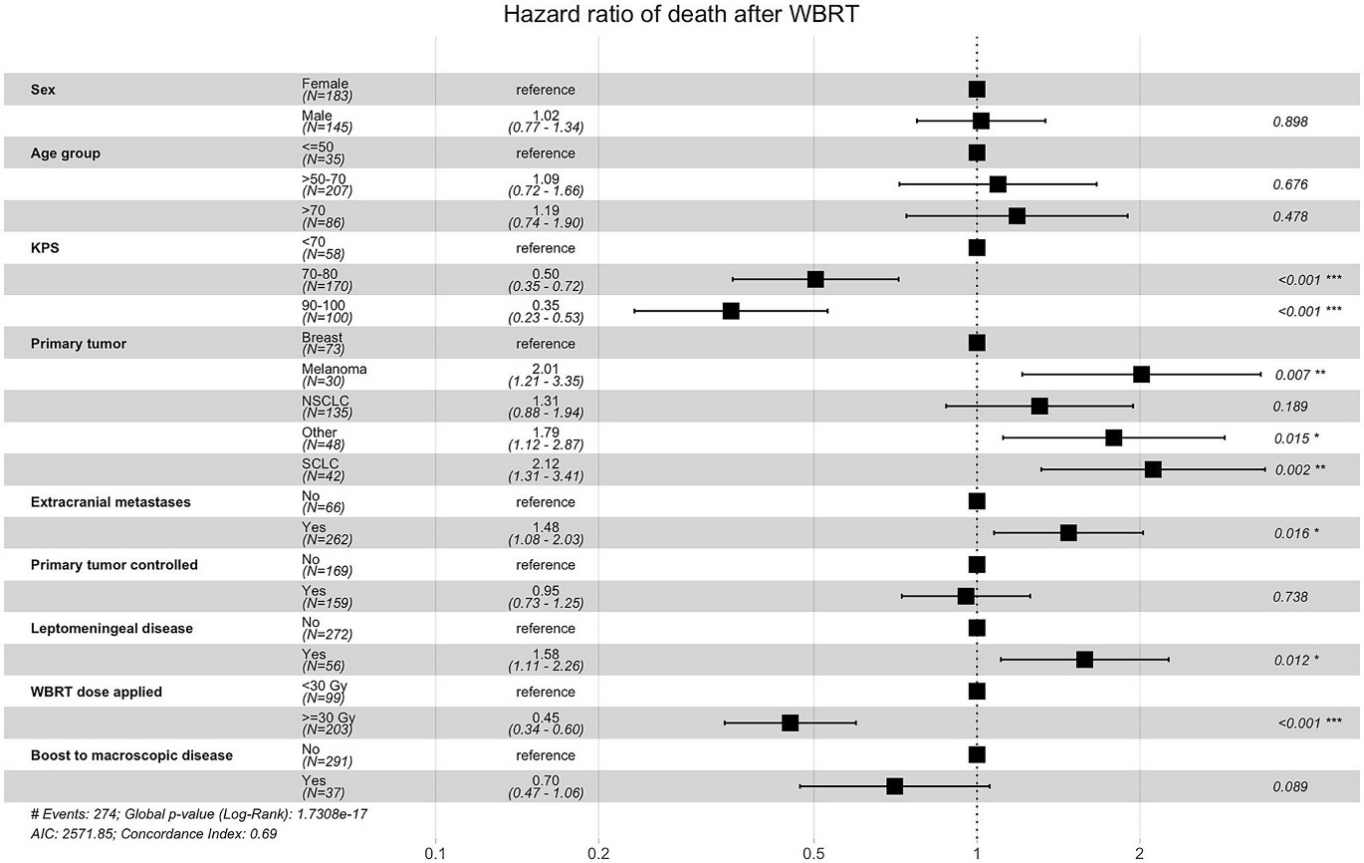
Multivariate Cox regression analysis showing the HR of death after WBRT in patients with brain metastases. *Abbreviations: HR* hazard ratio,* KPS* Karnofsky Performance Status,* NSCLC* non-small cell lung cancer,* SCLC* small cell lung cancer, *WBRT* whole brain radiation therapy

Results of the multivariate Cox regression analysis are shown in Fig. 2. Neither the patients’ sex nor age group were significant covariates for death after WBRT in our cohort. The covariate with the largest effect size was KPS, with a HR of 0.50 and 0.35 for patients with KPS 70–80 and 90–100, respectively (*p* < 0.001). The lowest HR of death was seen in patients with breast cancer (HR 1.0; reference), whereas patients with metastases from melanoma (HR 2.01; *p* = 0.007) and SCLC (HR 2.12; *p* = 0.002) appeared to have the worst outcomes. The presence of extracranial metastases (HR 1.48; *p* = 0.016) and leptomeningeal disease (HR 1.58; *p* = 0.012) was detrimental, whereas primary tumor control was not a significant factor (HR 0.95; *p* = 0.738) in the model. In patients who completed WBRT as scheduled (*n* = 302), those who received a dose of ≥ 30 Gy had a significantly lower HR of death (HR 0.45; *p* < 0.001) than those who had received < 30 Gy. Delivery of a boost to macroscopic disease was not associated with a lower HR of death, although a statistical trend could be observed (HR 0.70; *p* = 0.089).

**Fig. 3:**
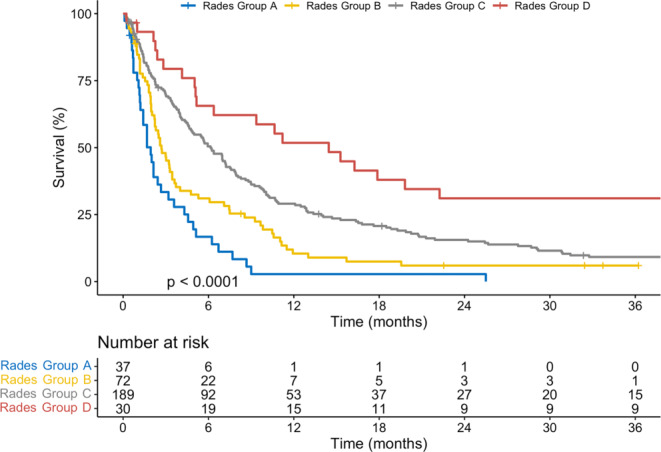
OS in patients classified according to the Rades scoring system, which was previously developed for patients undergoing WBRT for brain metastases. Survival outcomes differed between Rades subgroups in this more contemporary cohort, with a stepwise increase in OS seen from group A (poorest outcome) to D (best outcome). *Abbreviations: OS* overall survival,* WBRT* whole brain radiation therapy

Patients were reclassified according to the Rades scoring system, as described previously [20]. The KM estimates of OS for these different subgroups are shown in Fig. 3. Survival outcomes differed between subgroups based on the Rades scoring system (*p* < 0.01): median OS was 1.9 months (95%CI, 1.4–3.6), 2.7 months (95%CI, 2.2–3.6), 6.1 months (95%CI, 4.8–7.6) and 14.5 months (95%CI, 6.4–44.0) for groups A–D, respectively. The 6‑month survival rates for groups A–D were 17% (95%CI, 8–35%), 31% (95%CI, 22–44%), 50% (95%CI, 44–58%) and 66% (95%CI, 50–85%). For reference, the 6‑month survival rates in the original Rades cohort were 6%, 15%, 43% and 76% for groups A–D, respectively [20].

## Discussion

We report on the survival outcomes of patients undergoing WBRT for brain metastases in a contemporary setting. Overall, the survival outcomes appeared comparable to historical controls, reflecting the poor prognosis of patients with (multiple) brain metastases. However, we also observed significant differences between subgroups, and we identified prognostic factors which may assist clinical decision making in this setting.

Comparing outcomes of different cohorts is difficult due to a heterogeneity in patient selection, which may mask potential differences due to other factors, such as available treatment options. Our observation period of 2013–2021 covers a time during which the use of SRS has become more widespread, including in patients with multiple brain metastases. Furthermore, improvements in systemic therapies, including targeted agents with central nervous system (CNS) activity, have altered the treatment landscape for patients with brain metastases. In conjunction, these developments could have shifted the use of WBRT to later disease stages, when other approaches have failed and/or are no longer available. However, some patients with a very poor prognosis may have also been spared WBRT in the modern era. This may be particularly after 2016, when the QUARTZ trial suggested that WBRT offers little clinical benefit in NSCLC patients with brain metastases who are unsuitable for surgical resection or stereotactic radiotherapy [7]. The uncertainty surrounding WBRT outcomes in a contemporary era therefore led us to analyze our institutional outcomes in this cohort.

Overall, with a median OS of 4.7 months, our survival outcomes appear comparable to earlier WBRT series. A Japanese series of 111 patients (67% NSCLC) reported a median OS of 3.6 months after WBRT, and suggested that elevated lactate dehydrogenase (LDH) is an independent negative predictor for survival [22]. A Korean study used a large-scale health insurance claims database to identify patients who underwent radiotherapy for brain metastases, and reported a relatively long median OS of 8.4 months after WBRT, although most patients still died within the first year [23]. In contrast, median OS was only 2.5 months in a German series of 339 patients undergoing WBRT [9]. Different studies have sought to develop predictive models for survival, including a study from Thailand, which reported a median OS of 5.1 months in 389 patients who underwent WBRT for metastatic NSCLC [24]. We observed a similar median OS of 4.8 months in patients with NSCLC, which is notably better than the 2.1 months reported in QUARTZ, likely reflecting the selection of patients with a very poor prognosis for that trial [7, 12]. In addition, patients in QUARTZ received only 5 × 4 Gy, which may be considered a suboptimal dose based on our own observations.

Our analysis confirmed that a patient’s general condition, as measured by KPS, appears to be the most significant predictor for survival in patients undergoing brain radiotherapy, whereas age was not a significant factor in our cohort. Patients with brain metastases from breast cancer had the best prognosis, whereas extracranial metastases and leptomeningeal disease were prominent negative predictors. Notably, receipt of a WBRT dose of ≥ 30 Gy was a strong predictive factor in our model, indicating that these patients (typically treated with 10 × 3 Gy) had better survival than those who received < 30 Gy (typically 5 × 4 Gy). This is in contrast to the cohort of Rades et al., where there appeared to be no difference in survival between short-course and longer-course WBRT [20]. Although our multivariate analysis included a variety of prognostic factors, it is likely that additional confounders will have influenced the choice of fractionation regimens over time. Still, we consider it appropriate to offer longer-course WBRT in fit patients who may achieve longer-term survival (e.g. with controlled extracranial disease and/or effective systemic treatment options).

We confirmed that the Rades scoring system, based on WBRT data from 1992 to 2005, can still be used to classify patients into prognostic subgroups in a contemporary setting. However, the predictive accuracy for survival is likely very limited, considering the dramatic shifts in cancer care. Broadly, OS at 6 months appeared to be somewhat better in our cohort for Rades groups A–B, whereas this was overall similar for groups C–D. The Rades scoring system was developed exclusively for WBRT, whereas other prognostic scores have generally also included patients treated with surgery and/or SRS, in addition to other (methodological) differences that may limit their applicability to patients undergoing WBRT. For example, the updated DS-GPA excluded patients with recurrent brain metastases and/or leptomeningeal disease, which are common scenarios in which patients will undergo WBRT in a contemporary era [25]. In general, we observe that the acceptance of prognostic scores in daily clinical routine appears to be somewhat lacking. This may be due to the increasing complexity of individual clinical scenarios, for which prognostic indices may not be refined enough, particularly with regards to potential long-term survival [26, 27]. Additional work is therefore needed to develop tools for an era of personalized care, where patient-reported QoL could succeed OS as the primary outcome measure in patients undergoing WBRT.

Our study has several limitations. We focused on OS as the primary outcome measure, and did not report on the causes of death. This is similar to other reports in this setting, where the causes of death are often unknown due to limited diagnostics and/or patients dying at home or in hospice facilities. Similarly, the availability of follow-up imaging was variable, and additional studies are needed to explore patterns of relapse in our cohort. Due to the retrospective nature of the work, we cannot exclude that hidden confounders may have influenced results of our multivariate analysis. Furthermore, our study covered a period of approximately 8 years, during which significant changes in patterns of care may have occurred. Additional studies will therefore contribute to a more detailed understanding of individual patient outcomes, based on factors such as molecular alterations in different tumor subtypes, as well as receipt of systemic and/or additional local therapies.

## Conclusion

In conclusion, survival outcomes of patients undergoing WBRT in the contemporary era appear comparable to historical cohorts, although individual patient factors remain critical for clinical decision making. Patients with otherwise favorable prognostic factors (e.g. good performance status, controlled extracranial disease, effective systemic treatment options) may benefit from longer-course WBRT.

## References

[1] Gavrilovic IT, Posner JB (2005) Brain metastases: epidemiology and pathophysiology. J Neurooncol 75:5–14. 10.1007/s11060-004-8093-6 16215811 10.1007/s11060-004-8093-6

[2] Tsao MN, Xu W, Wong RKS et al (2018) Whole brain radiotherapy for the treatment of newly diagnosed multiple brain metastases. Cochrane Database Syst Rev. 10.1002/14651858.CD003869.pub4 29365347 10.1002/14651858.CD003869.pub4 PMC6491334

[3] Barnholtz-Sloan JS, Sloan AE, Davis FG, et al (2004) Incidence proportions of brain metastases in patients diagnosed (1973 to 2001) in the Metropolitan Detroit Cancer Surveillance System. J Clin Oncol Off J Am Soc Clin Oncol 22:2865–2872. 10.1200/JCO.2004.12.149 10.1200/JCO.2004.12.149 15254054

[4] Pease NJ, Edwards A, Moss LJ (2005) Effectiveness of whole brain radiotherapy in the treatment of brain metastases: a systematic review. Palliat Med 19:288–299. 10.1191/0269216305pm1017oa 15984501 10.1191/0269216305pm1017oa

[5] Stelzer KJ (2013) Epidemiology and prognosis of brain metastases. Surg Neurol Int 4:192–202. 10.4103/2152-7806.111296 10.4103/2152-7806.111296 PMC3656565 23717790

[6] Sperduto PW, Berkey B, Gaspar LE et al (2008) A New Prognostic Index and Comparison to Three Other Indices for Patients With Brain Metastases: An Analysis of 1,960 Patients in the RTOG Database. Int J Radiat Oncol Biol Phys 70:510–514. 10.1016/j.ijrobp.2007.06.074 17931798 10.1016/j.ijrobp.2007.06.074

[7] Mulvenna P, Nankivell M, Barton R, et al (2016) Dexamethasone and supportive care with or without whole brain radiotherapy in treating patients with non-small cell lung cancer with brain metastases unsuitable for resection or stereotactic radiotherapy (QUARTZ): results from a phase 3, non-inferiority, randomised trial. Lancet 388:2004–2014. 10.1016/S0140-6736(16)30825-X 10.1016/S0140-6736(16)30825-X PMC5082599 27604504

[8] Steindl A, Kreminger J, Moor E et al (2020) 363O Clinical characterization of a real-life cohort of 6001 patients with brain metastases from solid cancers treated between 1986–2020. Ann Oncol 31:397. 10.1016/j.annonc.2020.08.472

[9] Buecker R, Hong ZY, Liu XM et al (2019) Risk factors to identify patients who may not benefit from whole brain irradiation for brain metastases—A single institution analysis. Radiat Oncol 14:1–6. 10.1186/s13014-019-1245-9 30866972 10.1186/s13014-019-1245-9 PMC6417259

[10] Sperduto PW, Yang TJ, Beal K, et al (2017) Estimating Survival in Patients With Lung Cancer and Brain Metastases: An Update of the Graded Prognostic Assessment for Lung Cancer Using Molecular Markers (Lung-molGPA). JAMA Oncol 3:827–831. 10.1001/jamaoncol.2016.3834 10.1001/jamaoncol.2016.3834 PMC5824323 27892978

[11] Brown PD, Jaeckle K, Ballman K V, et al (2016) Effect of Radiosurgery Alone vs Radiosurgery With Whole Brain Radiation Therapy on Cognitive Function in Patients With 1 to 3 Brain Metastases: A Randomized Clinical Trial. JAMA 316:401–409. 10.1001/jama.2016.9839 10.1001/jama.2016.9839 PMC5313044 27458945

[12] Pechoux C Le, Dhermain F, Besse B (2016) Whole brain radiotherapy in patients with NSCLC and brain metastases. Lancet 388:1960–1962. 10.1016/S0140-6736(16)31391-5 10.1016/S0140-6736(16)31391-5 27604505

[13] Gaspar L, Scott C, Rotman M et al (1997) Recursive partitioning analysis (RPA) of prognostic factors in three Radiation Therapy Oncology Group (RTOG) brain metastases trials. Int J Radiat Oncol Biol Phys 37:745–751. 10.1016/s0360-3016(96)00619-0 9128946 10.1016/s0360-3016(96)00619-0

[14] Sperduto PW, Mesko S, Li J et al (2020) Beyond an Updated Graded Prognostic Assessment (Breast GPA): A Prognostic Index and Trends in Treatment and Survival in Breast Cancer Brain Metastases From 1985 to Today. Int J Radiat Oncol Biol Phys 107:334–343. 10.1016/j.ijrobp.2020.01.051 32084525 10.1016/j.ijrobp.2020.01.051 PMC7276246

[15] Sperduto PW, Chao ST, Sneed PK et al (2010) Diagnosis-specific prognostic factors, indexes, and treatment outcomes for patients with newly diagnosed brain metastases: a multi-institutional analysis of 4,259 patients. Int J Radiat Oncol Biol Phys 77:655–661. 10.1016/j.ijrobp.2009.08.025 19942357 10.1016/j.ijrobp.2009.08.025

[16] Sperduto PW, Mesko S, Li J et al (2020) Survival in Patients With Brain Metastases: Summary Report on the Updated Diagnosis-Specific Graded Prognostic Assessment and Definition of the Eligibility Quotient. J Clin Oncol 38:1–13. 10.1200/JCO.20.01255 32931399 10.1200/JCO.20.01255 PMC7655019

[17] Weltman E, Salvajoli JV, e Oliveira VC, et al (1998) Score Index for Stereotactic Radiosurgery of Brain Metastases. J Radiosurgery 1:89–97. 10.1023/B:JORA.0000010892.99686.9e

[18] Weltman E, Salvajoli J V, Brandt RA, et al (2000) Radiosurgery for brain metastases: a score index for predicting prognosis. Int J Radiat Oncol Biol Phys 46:1155–1161. 10.1016/s0360-3016(99)00549-0 10.1016/s0360-3016(99)00549-0 10725626

[19] Golden DW, Lamborn KR, McDermott MW, et al (2008) Prognostic factors and grading systems for overall survival in patients treated with radiosurgery for brain metastases: variation by primary site. J Neurosurg 109 Suppl:77–86. 10.3171/JNS/2008/109/12/S13 10.3171/JNS/2008/109/12/S13 19123892

[20] Rades D, Dunst J, Schild SE (2008) A new scoring system to predicting the survival of patients treated with whole-brain radiotherapy for brain metastases. Strahlentherapie Onkol 184:251–255. 10.1007/s00066-008-1831-5 10.1007/s00066-008-1831-5 18427755

[21] Rades D, Dziggel L, Haatanen T et al (2011) Scoring Systems to Estimate Intracerebral Control and Survival Rates of Patients Irradiated for Brain Metastases. Int J Radiat Oncol Biol Phys 80:1122–1127. 10.1016/j.ijrobp.2010.03.031 20638188 10.1016/j.ijrobp.2010.03.031

[22] Miyazawa K, Shikama N, Okazaki S et al (2018) Predicting prognosis of short survival time after palliative whole-brain radiotherapy. J Radiat Res 59:43–49. 10.1093/jrr/rrx058 29069502 10.1093/jrr/rrx058 PMC5778609

[23] Park K, Bae GH, Kim WK et al (2021) Radiotherapy for brain metastasis and long-term survival. Sci Rep 11:1–8. 10.1038/s41598-021-87357-x 33850188 10.1038/s41598-021-87357-x PMC8044241

[24] Trikhirhisthit K, Setakornnukul J, Thephamongkhol K (2022) Added survival benefit of whole brain radiotherapy in brain metastatic non-small cell lung cancer: Development and external validation of an individual prediction model. Front Oncol 12:1–10. 10.3389/fonc.2022.911835 10.3389/fonc.2022.911835 PMC9796174 36591469

[25] Sperduto PW, Mesko S, Li J et al (2020) Survival in Patients with Brain Metastases: Summary Report on the Updated Diagnosis-Specific Graded Prognostic Assessment and Definition of the Eligibility Quotient. J Clin Oncol 38:3773–3784. 10.1200/JCO.20.01255 32931399 10.1200/JCO.20.01255 PMC7655019

[26] Nieder C, Mehta MP (2009) Prognostic indices for brain metastases—usefulness and challenges. Radiat Oncol 4:10. 10.1186/1748-717X-4-10 19261187 10.1186/1748-717X-4-10 PMC2666747

[27] Hügel M, Stöhr J, Kuhnt T et al (2023) Long-term survival in patients with brain metastases—clinical characterization of a rare scenario. Strahlentherapie Onkol. 10.1007/s00066-023-02123-4 10.1007/s00066-023-02123-4 PMC10965568 37646818

